# Decomposition of changes in malaria prevalence amongst under-five children in Nigeria

**Published:** 2018-04-01

**Authors:** Deborah O. Owoeye, Joshua O. Akinyemi, Oyindamola B. Yusuf

**Affiliations:** 1 Department of Epidemiology and Medical Statistics, College of Medicine, University of Ibadan, Nigeria

## Abstract

**Background:**

Malaria remains a major public health problem in Nigeria. Changes in malaria prevalence can be attributed to three major control interventions: insecticide treated nets (ITNs), indoor residual spraying (IRS) and intermittent preventive treatment in pregnancy (IPTp). Use of ITNs has proven to be a practical, highly effective, and cost-effective intervention against malaria. Although, several studies have assessed the utilisation of ITNs, its impact on the prevalence of malaria over time is yet to be documented in Nigeria. Therefore, this study was conducted to decompose changes in malaria prevalence amongst under-five children between 2003 and 2013.

**Materials and methods:**

A retrospective analysis of the 2003 and 2013 Nigeria Demographic Health Survey (NDHS) dataset was conducted. Occurrence of fever was used as a proxy for malaria. Percentage change in both outcome and explanatory variables between 2003 and 2013 was estimated. A multivariate decomposition technique was used to partition changes in malaria prevalence into two components: contribution of changes in determinants and changes in the effect of determinants.

**Results:**

A total of 5204 and 28634 records of children under-five were available in 2003 and 2013 respectively. Malaria prevalence declined from 31.8% to 13.1% between 2003 and 2013 (p<0.001). Changes in determinants contributed 4.7% and changes in the effect of determinants contributed 95.3% to the change in malaria prevalence.

**Conclusions:**

There was a decline in the prevalence of malaria amongst children under five in Nigeria in the last ten years. Ownership of ITNs and their utilisation were the most contributing factors to the decline in malaria prevalence. Continued efforts should be made in promoting ITNs and their consistent and appropriate utilisation.

## 1 Introduction

Decomposition techniques are useful for identifying and explaining the separate contributions of group differences in measurable characteristics. Decomposition methods include, rate decomposition, the Oaxaca-Blinder means-coefficient decomposition, which allows coefficients to vary by subgroup, and quantile regression decomposition, which allows effects to vary across the distribution. The Oaxaca Blinder Decomposition (OBD) is a statistical method that explains the contribution of each factor to the difference in the outcome, thus identifying which factors contribute most to generating difference between the two groups [1]. Multivariate decomposition approach is applicable to many demographic outcomes, and it is especially useful for models that are nonlinear in parameters such as binary response like the presence and absence of a disease, event count, and hazard rate models [2].

Reports in medical literature suggest that OBD is suitable for decomposition of health data [3]. Oaxaca blinder decomposition has been applied to factors contributing to child mortality reductions in 142 low and middle-income countries between 1990 and 2010 [4]. Njau applied OBD to investigate the relationship between maternal education and childhood malaria infection rates in settings with relatively high malaria transmission; the study reported that there was a difference in malaria infection rates between children of educated mothers and those with mothers without education [5].

Malaria is endemic in Nigeria and constitutes a major public health problem. The disease overburdens the already-weakened health system: nearly 110 million clinical cases of malaria are diagnosed each year, and malaria contributes up to 60% of outpatient visits and 30% of admissions. Malaria also exerts a huge social and economic burden on families, communities, and the country at large, causing an annual loss of about 132 billion Naira in payments for treatment and prevention as well as hours not worked [6]. At least 50% of the population has at least one episode of malaria annually resulting in high productivity losses while children that are aged less than five years have 2-4 attacks annually [7].

The changes in malaria prevalence can be attributed to three major control interventions which are: insecticide treated nets (ITNs), indoor residual spraying (IRS) and intermittent preventive treatment in pregnancy (IPTp) [8]. African heads of states met in Abuja in April 2000 where they set among other targets in the Roll Back Malaria Program (RBM), a 60% use of ITNs among the high risk groups (pregnant women and under five children) by the year 2005 [9]. Since the launch of Roll Back Malaria initiative in Nigeria, ITNs distributed in 2005 is estimated to be 5 million (12 million since 2000). This has led to a substantial increase of household net ownership and ITN coverage rates in the 2003 estimates of 11.8% and 2.2%, respectively. Based on survey data collected between 2006 and 2007 the current national coverage of households with at least one ITN is estimated at 30-35% and that of ITN coverage at 10-15% [10]. Going by the sub-Saharan African standards, the use of ITNs in Nigeria is very low. The percentage of children under 5 in Nigeria who slept under ITNs increased only marginally from 3.3% in 2004 to 6% in 2008 [11]. There have been recent efforts to increase access to ITNs through mass distribution programmes but there are concerns that ITN utilisation may still lag behind which has been reported [12].

Despite the fact that ITN have been made available to the population, malaria still continues to be the major cause of child mortality and morbidity in Nigeria. People were knowledgeable about the benefits of using malaria preventive methods such as ITNs. But very few households spend money on malaria preventive tools. Also, the acquisition and usage of untreated mosquito nets was low for ITNs [13]. Although, several studies have assessed the utilisation of ITN [13-15], its impact on the prevalence of malaria over time is yet to be documented in Nigeria. Therefore, this study decomposed the changes in malaria prevalence among under-five children between 2003 and 2013 and estimated contribution of each determinant to changes in malaria prevalence.

## 2 Materials and methods

Data was extracted from the children dataset of the Nigeria Demographic Health Survey (NDHS 2003 and 2013). The sample size used for the analysis was 5204 in 2003 and 28634 in 2013. The inclusion criteria were children under-five years of age and women aged 15-49 years.

### 2.1 Study variables

The outcome variable was malaria. Occurrence of fever in the past two weeks was used as a proxy and was coded as: Yes ‘1’ and No ‘0’. The choice of explanatory variables was based on factors influencing occurrence of malaria as reported in the literature [16,17]: child’s age, sex of child, place of residence, mother’s education, wealth index, ITN ownership and use (i.e., children under-five that slept under an ITN the night before the survey).

### 2.2 Data analysis

Data was extracted from the NDHS 2003 and 2013 dataset, the data was cleaned and the missing values handled by using complete case analysis (listwise deletion) and weighted using SPSS version 20. Descriptive statistics, logistic regression and OBD were carried out using SPSS version 20 [18] and STATA 12 [19], respectively. Descriptive statistics (frequencies and proportions) was used to summarise malaria prevalence and it factors. Percentage change in both outcome and explanatory variables between 2003 and 2013 was estimated. A multivariate OBD decomposition technique was used to partition changes in malaria prevalence into two components: contribution of changes in determinants and changes in the effect of determinants.

Multivariate Oaxaca Blinder Decomposition: In Oaxaca blinder decomposition, regression model is fit for two groups, which are year 2003 and 2013. The regression model for the two groups was:


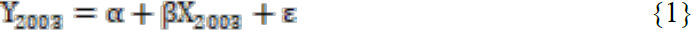



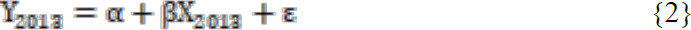


Where, Y_2003_ and Y_2013_ are the occurrence of malaria in year 2003 and 2013 respectively, α is an intercept term or constant, β is the coefficient of the explanatory variables and X_2003_ and X_2013_ are the vector of explanatory variables for year 2003 and 2013, respectively. ε is a residual term. The 2013 survey is assumed to have a higher mean of Y and the 2003 have a lower mean value of Y than 2013. The change between the mean outcomes of the two groups is given by:


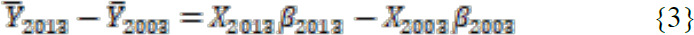


This expression can in turn be written as the sum of the following three terms:


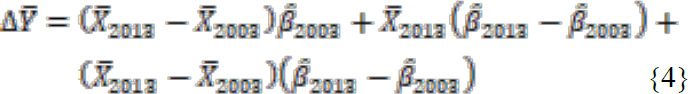


The first term on the right-hand side of equation 4 is the explained part called ‘decomposition effect’ or ‘endowment term’, the second term and the third term are the unexplained part called ‘coefficient term’ and “interaction term” respectively. The endowments term represents the contribution of differences in explanatory variables across groups, and the coefficients term is the part that is due to group differences in the coefficients. Finally, the interaction term accounts for the fact across group differences in explanatory variables and coefficients can occur at the same time [20].


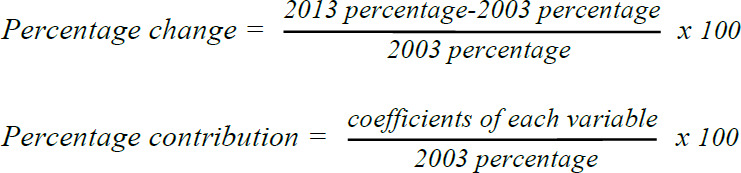


## 3 Results

Table 1 shows the change in malaria prevalence according to selected background characteristics. Childhood malaria was more prevalent among children aged 12-23 months (25.5% in 2003 and 28.4% in 2013). This was followed by children aged 0-11months (25.6% in 2003 and 21.5% in 2013) with a decrease in the prevalence of malaria by 17.2% over the period of ten years. Children aged 48-59 months had the lowest occurrence of malaria (11.6% in 2003 and 13.2% in 2013). Prevalence of malaria was high amongst children whose mother had no formal education (53.2% in 2003 and 46.4% in 2013) with a 12.8% decrease between the two years. While there was an increase of 36.9% in malaria prevalence among children whose mothers had secondary/higher education (23.6% in 2003 and 32.3% in 2013). Malaria was most prevalent amongst children in rural areas (68.2% in 2003 and 68.2% in 2013) with no change between the two years. About one-quarter (25.2% in 2003 and 24.2% in 2013) of children in the poorest wealth quintile had malaria compared to their counterparts in the richest wealth quintile (13.0% in 2003 and 12.2% in 2013) which yielded a 6.2% decrease in malaria prevalence.

**Table 1:** Change in malaria prevalence between 2003 and 2013 according to selected background characteristics.

Factor	Prevalence in 2003 (%)	95% CI		Prevalence in 2013 (%)	95% CI		% Change
		Upper	Lower		Upper	Lower	
*Age (Months)*							
0-11	411 (25.6)	1.795	1.873	783 (21.2)	1.914	1.948	-17.2
12-23	409 (25.5)			1049 (28.4)			11.4
24-35	330 (20.6)			748 (20.3)			-1.5
36-47	267 (16.7)			622 (16.9)			1.2
48-59	186 (11.6)			489 (13.2)			13.8
*Sex*							
Male	818 (51.0)	1.479	1.507	1902 (51.5)	1.491	1.503	1.0
Female	785 (49.0)			1789 (48.5)			-1.0
*Maternal education*							
No education	852 (53.2)	0.762	0.809	1711 (46.4)	0.871	0.892	-12.8
Primary	373 (23.3)			786 (21.3)			-8.6
Secondary/ Higher	378 (23.6)			1194 (32.3)			36.9
*Residence*							
Rural	1093 (68.2)	1.619	1.646	2518 (68.2)	1.655	1.666	0
Urban	510 (31.8)			1173 (31.8)			0
*Wealth index*							
Poorest	404 (25.2)	2.861	2.939	892 (24.2)	2.839	2.872	-4.0
Poorer	358 (22.3)			912 (24.7)			10.8
Middle	324 (20.2)			807 (21.9)			8.4
Richer	309 (19.3)			629 (17.0)			-11.9
Richest	208 (13.0)			451 (12.2)			-6.2
*Ownership of ITN*	243 (15.2)	0.129	0.149	2593 (70.3)	0.669	0.681	362.5
*Utilization of ITN*	114 (7.5)	0.063	0.077	819 (22.5)	0.219	0.229	212.5

**Figure 1:**
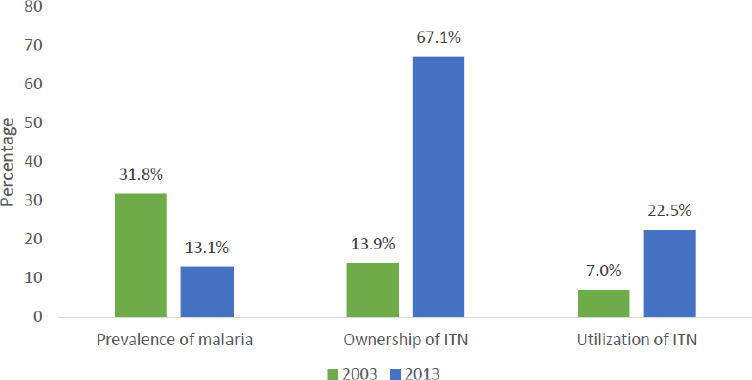
Percentage in prevalence of malaria and ITN.

Figure 1 shows the percentage in prevalence of malaria and ITN. Childhood malaria was more prevalent in 2003 (31.8%) than in 2013 (13.1%). This translates into a 59% decrease in prevalence within a decade. There was a large increase in the ownership of ITNs between the two years (383%). Similarly, utilisation of ITNs increased by approximately 221% from 7.0% in 2003 to 22.5% in 2013.

To investigate the contribution of selected factors to the difference in prevalence the change thereof was decomposed into three parts: The first part is known as the decomposition effect which is due to differences in the distribution of the determinants between year 2003 and 2013. The second part is the coefficient effect which is the part due to differences in the effects of these determinants between the groups, and the third part is the interaction effect which is the interaction between decomposition effect and coefficient effect. In Table 2, the overall decomposition model showed that prevalence of malaria was 0.319 (95% CI= 0.306, 0.332) in year 2003 and 0.131 (95% CI= 0.127, 0.135) in year 2013 with a difference of 0.188 (95% CI= 0.174, 0.201). Table 2 also shows the difference of each determinant between year 2003 and 2013, which accounted for 4.7% of the observed reduction in malaria (95% CI= -0.014, -0.005) and the change in impact of each determinant (coefficient) accounted for approximately 95.3% of the decrease in malaria (95% CI= 0.174, 0.225) over the period of ten years. The interaction effect was marginal and not significant.

**Table 2:** Overall decomposition model results of malaria prevalence amongst children under-five between 2003 and 2013.

	Coefficient	SE	95% CI		P-value	% Contribution
			Lower	Upper		
2003 Survey	0.319	0.007	0.306	0.332	<0.001	
2013 Survey	0.131	0.002	0.127	0.135	<0.001	
Difference	0.188	0.007	0.174	0.201	<0.001	
Determinants	0.001	0.002	-0.014	-0.005	<0.001	4.7
Coefficients	0.199	0.013	0.174	0.225	<0.001	95.3
Interaction	-0.001	0.011	-0.024	0.021	0.896	

SE = Standard Error; CI= Confidence Interval

Table 3 shows the contribution of each determinant to the change in malaria over time. The positive percentages show a positive contribution to the difference on malaria between year 2003 and 2013. Ownership of ITN was the largest factor (92%) explaining the difference in malaria prevalence between the two years (95% CI= -0.016, -0.006). Also, utilisation of ITN accounted for 13.3% of the observed reduction in malaria (95% CI= 0.001, 0.003).

**Table 3:** Contribution of determinants of malaria prevalence amongst children under-five between 2003 and 2013.

Factors	Coefficient	SE	95% CI		P-value	% Contribution
			Lower	Upper		
*Age*	0.00114	0.00029	0.0005	0.0017	<0.001	9.7
*Sex*						
Male*						
Female	0.00001	0.0001	-0.0001	0.0001	0.789	0.1
*Residence*						
Urban*						
Rural	0.0005	0.00019	0.0001	0.0008	0.013	4.0
*Maternal education*						
None*						
Primary	0.0007	0.00024	0.0002	0.0012	0.004	5.9
Secondary/Higher	-0.0016	0.00040	-0.0024	-0.0008	<0.001	-14.0
*Wealth index*						
Poorest*						
Poorer	0.00014	0.00012	-0.0001	0.0004	0.218	1.2
Middle	0.00008	0.00011	-0.0001	0 .0003	0.457	0.7
Richer	-0.0005	0.00031	-0.0011	0.0001	0.099	-4.3
Richest	-0.0001	0.00048	-0.0019	-0.0001	0.043	-8.3
*Ownership of ITN*						
No*						
Yes	0.0107	0.00242	-0.0155	-0.0059	<0.001	91.7
*Utilisation of ITN*						
No*						
Yes	0.0016	0.000775	0.00003	0.0031	0.045	13.3
Total	0.01168					100

SE = Standard Error; CI= Confidence Interval; * = Reference category

In the observed reduction in malaria, child’s age accounted for 9.7% (95% CI= 0.001, 0.002) but the child’s sex had the lowest contribution of 0.1% (95% CI= -0.001, 0.001). Residence contributed approximately 4% to the observed reduction (95% CI= 0.000, 0.001). Maternal primary education contributed 5.9% to the difference (95% CI= 0.000, 0.001), while higher maternal education contributed 14.0% (95% CI= -0.002, -0.001) to the observed difference in malaria between the two years. Wealth index accounted for -10.7% of the difference in malaria with children in the poorer wealth quintile contributing 1.2%, while, children in the middle wealth quintile contributed 0.7%, children in the richer wealth quintile contributed -4.3% and children in the richest wealth quintile contributed -8.3% (95% CI= - 0.002, -0.001) to the observed reduction.

## 4 Discussion

Our findings revealed that malaria prevalence declined between 2003 and 2013. This decline is not surprising when considering the huge resource investments and interventions put forward by the RBM, NMEP (national malaria eradication programme) [21]. Several studies have documented reductions in malaria prevalence in African countries [22-25]. In a study in Senegal, there was a decrease of 32% of malaria between 1996 and 2010 [26]. Also, in Malawi, the proportion of malaria cases decreased by 50% between 2001 and 2005 [27]. A study reported that malaria was reduced by almost 85% in two communities in Tanzania [28].

The current study revealed a decline in the prevalence of malaria, but malaria nevertheless remains highly prevalent in Nigeria [29]. A study in the northern part of Nigeria revealed a prevalence of 56.9% of malaria among under-five children in 2008 [30]. In that study, children whose mother had no formal education had a higher malaria prevalence, which may be associated to the fact that children were more likely to receive prompt and effective treatment if their mothers had at least a secondary education. A study documented that mothers that are educated may be open to advances in public health and medicine, more aware of malaria symptoms and related health risks [31]. Also, studies have shown that there is a correlation between maternal education and child health [32] as mothers who are educated tend to be more knowledgeable about health problems and how to deal with these. Likewise, another study reported that the more educated a mother is, the less likely for the child to have malaria and this may be due to the fact that an educated mother will understand information on malaria better and is more likely to implement the preventive measures she has been taught [33].

The results of the decomposition also showed that there is a significant decline in the prevalence of malaria amongst children under five in 2013 than in 2003. This was similar to several other studies which reported declines in malaria prevalence [23,26]. The results also showed that the decline is mainly due to changes in the effect of the determinants (coefficient parts). The analysis revealed that the key factors that determined the prevalence of malaria are ITN ownership and ITN utilization. This was in agreement with other studies which reported an association between utilisation of ITNs and malaria prevalence [34]. Undoubtedly, these variables have remained important factors to consider in implementing policies to further reduce the prevalence of malaria. Since fever is a symptom for many childhood illnesses and not always tests were performed to confirm malaria it is possible that false-positives were included in the data.

## 5 Conclusions

In Nigeria, a significant decline in malaria prevalence has been observed between 2003 and 2013. ITN ownership and utilisation were the most contributing factors to the observed decline. More efforts should be made to promote ITN usage as well as wide-scale free net distribution.
